# Neural Entrainment to Polyrhythms: A Comparison of Musicians and Non-musicians

**DOI:** 10.3389/fnins.2017.00208

**Published:** 2017-04-12

**Authors:** Jan Stupacher, Guilherme Wood, Matthias Witte

**Affiliations:** ^1^Department of Psychology, University of GrazGraz, Austria; ^2^BioTechMed-GrazGraz, Austria

**Keywords:** music cognition, rhythm perception, entrainment, temporal prediction, neural oscillations, steady-state evoked potentials, EEG, musical training

## Abstract

Music can be thought of as a dynamic path over time. In most cases, the rhythmic structure of this path, such as specific sequences of strong and weak beats or recurring patterns, allows us to predict *what* and particularly *when* sounds are going to happen. Without this ability we would not be able to entrain body movements to music, like we do when we dance. By combining EEG and behavioral measures, the current study provides evidence illustrating the importance of ongoing neural oscillations at beat-related frequencies—i.e., neural entrainment—for tracking and predicting musical rhythms. Participants (13 musicians and 13 non-musicians) listened to drum rhythms that switched from a quadruple rhythm to a 3-over-4 polyrhythm. After a silent period of ~2–3 s, participants had to decide whether a target stimulus was presented on time with the triple beat of the polyrhythm, too early, or too late. Results showed that neural oscillations reflected the rhythmic structure of both the simple quadruple rhythm and the more complex polyrhythm with no differences between musicians and non-musicians. During silent periods, the observation of time-frequency plots and more commonly used frequency spectra analyses suggest that beat-related neural oscillations were more pronounced in musicians compared to non-musicians. Neural oscillations during silent periods are not driven by an external input and therefore are thought to reflect top-down controlled *endogenous* neural entrainment. The functional relevance of *endogenous* neural entrainment was demonstrated by a positive correlation between the amplitude of task-relevant neural oscillations during silent periods and the number of correctly identified target stimuli. In sum, our findings add to the evidence supporting the neural resonance theory of pulse and meter. Furthermore, they indicate that beat-related top-down controlled neural oscillations can exist without external stimulation and suggest that those endogenous oscillations are strengthened by musical expertise. Finally, this study shows that the analysis of neural oscillations can be a useful tool to assess how we perceive and process complex auditory stimuli such as polyrhythms.

## Introduction

Temporal aspects of music—the organization of sounds into patterns and the unfolding of those patterns over time—allow us to predict what and when sounds are likely to occur next. It is supposed that such expectations and predictions are crucial for the experience of pleasure during music listening (Salimpoor et al., 2015). Thus, the question of how we track the temporal structure of music is key for understanding why we love music.

On a basic level, *musical rhythms* can be defined as acoustic sequences with patterns of duration and accentuation; they represent the structure of music (London, 2004; Large et al., 2015). We can tap our feet or bob our heads in time with music because we are usually able to perceive a regular *beat* or *pulse* within a rhythm. If we perceive strong and weak beats, detect regularly recurring events, and use these events to group a fixed number of beats in cycles, we can be said to have found the *meter* of a musical piece (London, 2004). Broadly speaking, a quadruple meter encourages us to tap our feet four times (beat-level) in each cycle (bar-level), whereas a triple meter encourages us to tap three times in each cycle.

*Polyrhythms* consist of at least two different beat-levels (N and M) within one bar-level, whereby N and M have to be relatively prime (i.e., they have no common roots; Large and Kolen, 1994). Polyrhythms, such as 3:2, 4:3, 5:4, or 7:5, create rhythmic tension and are used in different genres including, but not limited to jazz (Pressing, 2002), rock, metal (Pieslak, 2007; Osborn, 2010), African drumming (Locke, 1982), and twentieth century Western art music (Poudrier and Repp, 2013). Described from a perspective of embodiment, polyrhythms can be seen as contrasting but coordinated body movements (Iyer, 2002). For example, let us imagine a 3:2 polyrhythm. A dancer listening to this polyrhythm might stamp his or her feet three times each cycle, aligned with the triple beat, while in the same amount of time moving his or her upper body once to the left and once to the right in time with the duple beat.

Various bimanual tapping studies predominantly conclude that contrasting pulse trains in simple polyrhythms are not processed by parallel timekeepers, but integrated into one shared rhythmic framework (e.g., Klapp, 1979; Deutsch, 1983; Klapp et al., 1985, 1998; Jagacinski et al., 1988; Summers and Kennedy, 1992; Summers et al., 1993; Peper et al., 1995; Krampe et al., 2000; Kurtz and Lee, 2003). However, at least in professional musicians, bimanual tapping of faster polyrhythms might be controlled by independent timekeepers (Krampe et al., 2000). Even without overt movements, integrated processing of contrasting patterns can be beneficial when tracking polyrhythms (e.g., Jones et al., 1995; Klein and Jones, 1996). Jones et al. (1995) examined attentional strategies of musicians and non-musicians when trying to detect timing changes of target tones in 3:2 polyrhythms. In a series of experiments that encouraged either integrative or selective attending, the authors found that although musicians as compared to non-musicians were more accurate in detecting timing changes, they were not better at adjusting their attentional strategy (i.e., choosing integrated vs. selective attending).

Whereas, most of the aforementioned studies used simple polyrhythms consisting of two contrasting isochronous pulse trains, Poudrier and Repp (2013) examined whether classically trained musicians use divided or selective attention when keeping track of polyrhythms of different complexities in a perceptual probing paradigm. In simple polyrhythms the two contrasting parts (2/4 and 6/8 meter) could be tracked simultaneously. However, more complex polyrhythms limited the musicians' ability to divide their attention. Keller and Burnham (2005) found similar results in a dual-task paradigm that examined the recognition and reproduction of matched-metrical and mismatched-metrical rhythmic patterns. In a recent study, Wöllner and Halpern (2016) asked expert and novice conductors and pianists to detect pitch and timing deviations of target tones in selective- and divided-attention tasks (focus on one of two melodic streams, and focus on both streams, respectively). They found that in both tasks experts detected more targets than students. Furthermore, conductors—specialists in monitoring multiple parts of a musical piece simultaneously—detected more targets than pianists in the divided-attention task.

Training-related differences were also shown in sensorimotor synchronization studies. Finger tapping studies show that musical training can sharpen rhythm perception and production (e.g., Chen et al., 2008; Cameron and Grahn, 2014; Matthews et al., 2016). Another tapping study by Drake et al. (2000b) suggests that musicians as compared to non-musicians have a better apprehension of metric structure and can process a wider range of hierarchical levels. These behavioral results are supported by EEG studies examining event-related potentials (ERPs) elicited by target tones within musical rhythms. In isochronous sequences of unaccented tones that typically induce binary subjective accenting (“tick”–“tock”–“tick”–“tock”…), ERPs differed more strongly between deviants that fell on subjectively strong and subjectively weak beats for musicians as compared to non-musicians (Brochard et al., 2003). Another study found that, in musicians but not in non-musicians, mismatch negativities (MMN: an ERP difference measure that is increased by unexpected stimuli) were elicited by violations of numerical regularity in tone sequences (van Zuijen et al., 2005). Additionally, the difference between MMNs elicited by meter-congruent and meter-incongruent target tones was larger in musicians as compared to non-musicians (Geiser et al., 2010).

The enhanced ability of musicians in the processing of hierarchical temporal structures might relate to an increased involvement of the working memory, as reflected by an enhanced recruitment of the dorsolateral prefrontal cortex (Chen et al., 2008). Musicians' advantages in rhythm perception and performance can be linked to the tight coupling between sensory and motor areas of the brain that develops through musical training and experience (Haueisen and Knösche, 2001; Bangert and Altenmüller, 2003; Bangert et al., 2006; D'Ausilio et al., 2006; Grahn and Rowe, 2009; Stupacher et al., 2013). Although, previous research has shown differences between musicians and non-musicians in sensorimotor synchronization, perceptual organization of rhythm, and neural processing, it remains an open question whether rhythm-related neural oscillations are modulated by musical expertise and in which ways (Nozaradan, 2014).

Neural oscillations are based on synchronized fluctuations between high and low excitability states in groups of neurons (Singer, 1999; Fries, 2005), a function that is, among others, important for attention and prediction (Buzsáki and Draguhn, 2004; Schroeder and Lakatos, 2009; Arnal and Giraud, 2012; Frey et al., 2015). In music, meter describes the percept of a regular temporal structure with alternating strong and weak events. Resonance theories link pulse and meter perception with neural oscillations (Large, 2008). They assume that when neural oscillations *entrain* to a stimulus with at least some temporal regularity, such as most music, high neural excitability states and the underlying pulse of the musical rhythm become aligned (Large and Kolen, 1994; Large and Jones, 1999; Large, 2008; Schroeder and Lakatos, 2009). When measured with EEG, such entrained oscillations are called steady-state evoked potentials (SSEP) and are reflected in amplitude peaks in the frequency spectra of ongoing signals. In a series of studies Nozaradan and colleagues showed that beat-related SSEPs are influenced by the imagination of accents within unaccented isochronous pulse trains (duple vs. triple meter imagery; Nozaradan et al., 2011), are present with rhythmic patterns whose envelope spectra do not necessarily have maximum peak amplitudes at the beat frequency (Nozaradan et al., 2012a), and relate to sensorimotor coupling (Nozaradan et al., 2015). Whereas, Nozaradan and colleagues used abstract and relatively simple auditory stimuli, Tierney and Kraus (2015) showed that neural entrainment also plays a key role in tracking the rhythm of real music.

In a previous experiment we used pop/rock drum rhythms and isochronous pulse trains to show that, during listening, stimuli with more complex rhythmic hierarchies, as compared to isochronous stimuli, elicited more pronounced SSEPs at the beat's second harmonic frequency (Stupacher et al., 2016). In the same experiment, auditory stimuli were interrupted by silent breaks (2–6 s), allowing us to examine whether neural oscillations persist without external stimulation as well as to connect ongoing neural oscillations (SSEPs) and transient neural responses (ERPs). Similar to the SSEP results, the N1 ERP component indicated that listening to more complex rhythms increased neural entrainment. Although, we were not able to *directly* show neural oscillations in the frequency domain during silent breaks, N1 results suggested that beat-related oscillations persist throughout the breaks, but become blurred and/or decrease in their amplitude with increasing time. This is an important finding, as it suggests that beat-related neural oscillations are not merely a reflection of the auditory stimulus (bottom-up processing), but rather involve top-down endogenous timing processes.

The silent break design indirectly instructed the participants to imagine that the rhythm continued during silence. Gordon (1985, 1999) coined the term *audiation*, which describes the hearing of music in the mind. Audiation involves the interpretation and understanding of what was just heard, recalled, composed or performed. Several neuropsychological studies indicate that the auditory cortex can be active without the actual presence of sound and suggest that musical imagery is related to this activity (Zatorre and Halpern, 2005). Audiation, or more generally musical imagery, is an important part of music education (e.g., Gordon, 1999). A recent study showed that musical training and higher musical engagement were associated with better performance in a musical tempo imagery task (Jakubowski et al., 2016).

In the current experiment, we decided to adapt the silent break design of Stupacher et al. (2016). Musicians and non-musicians listened to auditory stimuli that started with an isorhythmic quadruple drum rhythm and turned into a 3-over-4 (4:3) polyrhythm. After the polyrhythm, we included a silent period of ~2 to 3 s after which a target stimulus was presented. Participants had to decide whether this target stimulus fell on the timing grid of the triple-beat part of the 4:3 polyrhythm or was presented 220 ms too early or 220 ms too late. This design allowed us to address various open questions: How do neural oscillations evolve during listening to stimuli with changing metrical hierarchies? Do neural oscillations persist without an acoustic input when the task demands to imagine that the beat goes on? And does musical expertise modulate neural entrainment?

## Methods

### Participants

Twenty-six adults (11 female; mean age = 26.8 years, *SD* = 7.07) participated in the experiment. One additional participant was excluded due to an unacceptable amount of noise in the EEG recording. Thirteen participants (6 female), hereafter referred to as *musicians*, had played their primary instrument (including drums/percussion, piano, guitar, bass, and violin/viola) for *M* = 15.5 years (*SD* = 3.71). The average amount of formal training on their primary instruments was 11.8 years (*SD* = 5.13). The starting age of the musicians ranged from 5 to 12 years (*M* = 7.9 years, *SD* = 2.43). Seven musicians played a second instrument (*M* = 8.8 years, *SD* = 5.00), and two musicians played a third instrument (4 and 9.5 years). Ten musicians played in ensembles for *M* = 7.2 years (*SD* = 4.44). The remaining 13 participants, hereafter referred to as *non-musicians*, had no musical training. Participants gave written informed consent in accordance with the Declaration of Helsinki and were paid for their participation. The study was approved by the ethics committee of the University of Graz.

### Auditory stimuli

**Figure 1:**
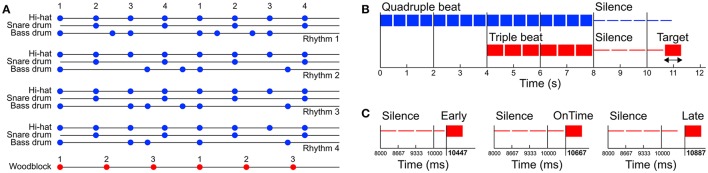
**(A)** Structure of the four quadruple rhythms consisting of hi-hat, snare drum, and bass drum (blue dots, Rhythms 1–4) and the triple beat consisting of a woodblock sound (red dots). Numbers represent the beat count. **(B)** Structure of one trial. Before the stimuli started two metronome clicks were presented at the same rate as the quadruple beat (−1,000 and −500 ms). The trials were composed of one of the four quadruple rhythms (0–4 s), followed by a 4:3 polyrhythm including the triple-beat woodblock (4–8 s). After a subsequent silent period with varying duration a target stimulus was presented. **(C)** The target stimulus was either presented directly on the timing grid of the cycles second triple beat (*on time*, mid panel), 220 ms too *early* (left panel), or 220 ms too *late* (right panel). Note that the second triple beat does not coincide with a quadruple beat.

**Figure 2:**
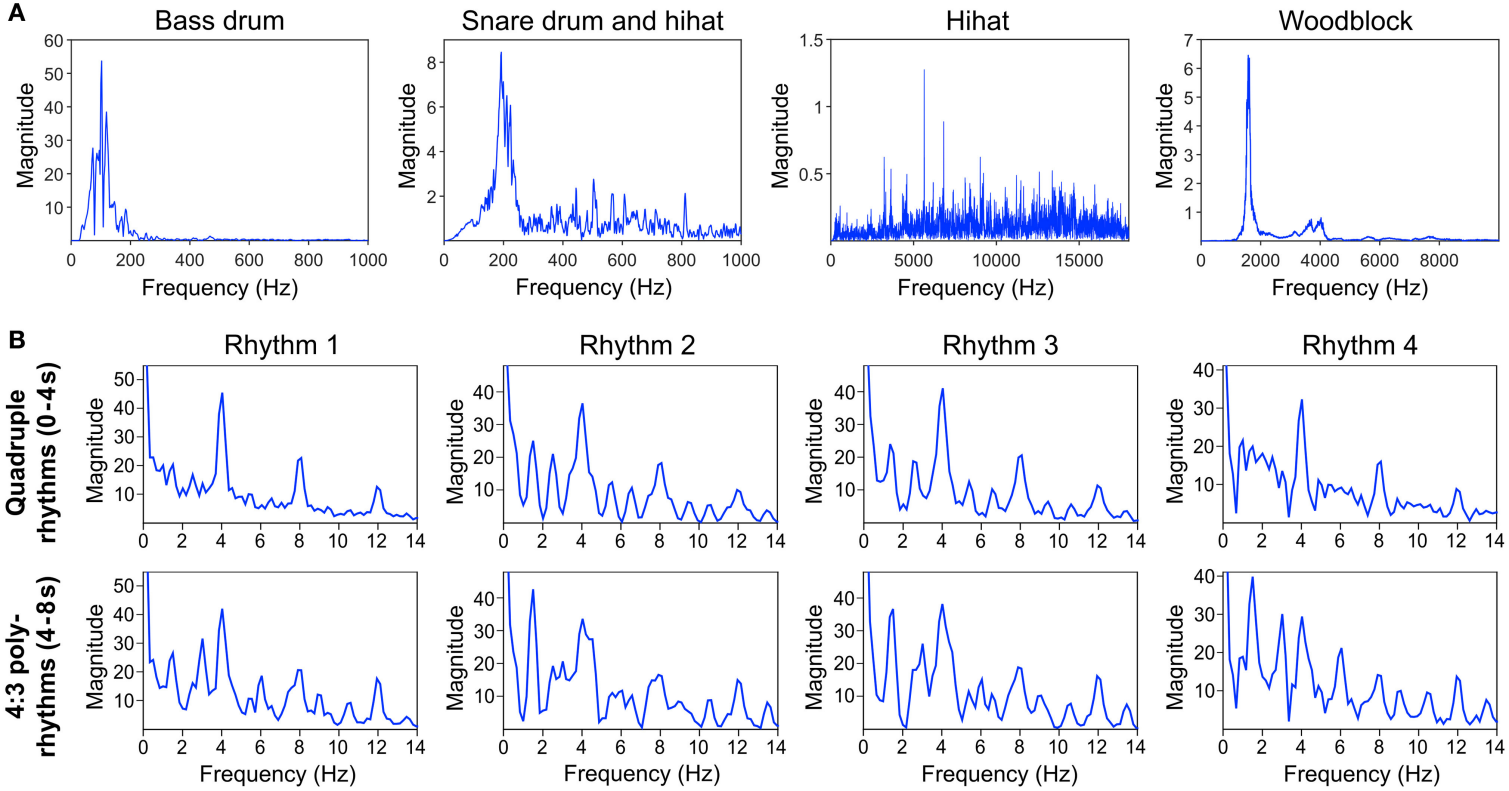
**(A)** Frequency spectra of the different instruments' *audio waveforms*. **(B)** Frequency spectra of the *sound envelopes* of quadruple rhythm parts (0–4 s, top panel) and corresponding 4:3 polyrhythm parts (4–8 s, bottom panel) of the four different rhythms depicted in Figure 1A. Envelopes and spectra were computed with *mirenvelope* and *mirspectrum* of the MIR toolbox 1.6 for Matlab (Lartillot and Toiviainen, 2007).

Auditory stimuli were programmed with the software Ableton Live (version 8; Ableton AG, Berlin, Germany) and consisted of four different basic pop/rock drum rhythms played by bass drum, snare drum and hi-hat (Figure 1A and Supplementary Material). Variations of the drum rhythms were subtle and only applied to the bass drum track. The hi-hat and snare drum tracks were identical in all four drum rhythms. The rhythms were created in quadruple meter (1 – 2 – 3 – 4 – / 1 – 2…) at a tempo of 120 beats per minute (BPM) and lasted four bars (i.e., four cycles of four beats, 8 s in total). The tempo was chosen because previous research shows that 120 BPM (2 Hz) is an intuitive and comfortable rate for sensorimotor synchronization (van Noorden and Moelants, 1999; Drake et al., 2000a; MacDougall and Moore, 2005; Repp, 2005). After two bars (4 s) a woodblock sound with three evenly distributed tone onsets per bar (1 – 2 – 3 – / 1 – 2…) was added to the isorhythmic drum rhythms, resulting in a simple 4:3 polyrhythm (Figure 1B). This isochronous triple woodblock beat was the same for each of the four quadruple drum rhythms. The volume and timbre of the woodblock sound was optimized to make it clearly audible and distinguishable, as previous research suggests that larger pitch separations between two contrasting parts of a polyrhythm make it easier to focus on a single part and make an integrated representation of the two parts less likely (Klapp et al., 1985; Moelants and van Noorden, 2005; Fidali et al., 2013). Figure 2A shows the frequency spectra of the audio waveforms of the different instruments.

Quadruple drum rhythms were quantized on an eighth note level (250 ms grid), whereas the triple woodblocks were quantized on a 666.67 ms grid (exactly 3 onsets per bar). The two bars of quadruple rhythm and the subsequent two bars of 4:3 polyrhythm were followed by a silent period of four triple notes (1 – 2 – 3 – / 1 –). After this silent period a target stimulus was presented with the same woodblock sound as in the 4:3 polyrhythm. The target stimulus was either perfectly in time with the second triple beat, 220 ms too early, or 220 ms too late (Figure 1C). We chose to present the target stimulus on the second beat of the triple meter because the first triple beat would have matched with the first quadruple beat (Figure 1) and the task of the participants was to focus on the triple beat (see Design and Procedure Section below).

Figure 2B shows the frequency spectra of the sound envelopes of the four quadruple drum rhythms (first 4 s of each trial; top panel) and the corresponding frequency spectra of the 4:3 polyrhythms (4–8 s of each trial; bottom panel).

To make it easier for the participants to “get into the beat,” and to allow some time for beat-related neural oscillations to evolve, two clicks were presented at the quadruple beat rate before the rhythms started (−1,000 and −500 ms relative to the first onset of the rhythms).

### Design and procedure

Participants sat in a cushioned chair with an attached table on which they could rest their arms. They were instructed to sit relaxed and to avoid general movements and movements in time with the musical rhythms. To monitor body movements, electromyography (EMG) signals were recorded from the neck (splenius capitis), both arms (extensor digitorum communis), and both legs (musculus tibialis anterior) using Ag/AgCl electrodes and a ground electrode on the clavicle.

Auditory stimuli were presented with the software Presentation (version 17; Neurobehavioral Systems, Berkeley, CA), an ART HeadAmp 4 headphone amplifier (Art Pro Audio, Niagara Falls, NY), and Aircom A5 Airtube in-ear headphones (Aircom Audio Inc., Brea, CA). Twelve different trials resulted from the combination of the four different rhythms and the three different positions of the target stimulus (220 ms too early, on time, or 220 ms too late). Participants were presented 10 blocks with all 12 trials, resulting in 120 trials in total.

The task was explained in an instruction video that showed how the auditory stimuli were structured and included an example stimulus. Participants were instructed to focus on the triple beat of the 4:3 polyrhythm, to try to imagine how this beat continues, and to decide whether the target stimulus after the silent period was exactly on time with the triple beat, too early, or too late. The answer was given on a computer keyboard with the left arrow key for targets that were too early, the up arrow key for targets on time, and the right arrow key for targets that were too late. During the presentation of the stimuli a fixation cross was displayed on a computer monitor 1.5 m in front of the participants. One second after the target stimulus was presented, participants were asked to make their decision. As soon as they pressed one of the arrow keys the next trial started with a delay of 2 s. After each block of 12 trials, participants had the chance to take a break and to continue the next block by pressing the space bar. To ensure that participants understood the task, five practice trials were observed by the experimenter.

At the beginning and at the end of the experiment, participants additionally listened to a 2-min recording of the woodblock sound with a 666.67 ms/1.5 Hz inter-onset-interval (i.e., the same stimulus that was used to create the 4:3 polyrhythms in combination with the quadruple drum rhythms). Hereafter we refer to this metronome as *triple metronome*.

### EEG recording

Fifteen Ag/AgCl electrodes were placed on the scalp according to the international 10-20 system to record the electroencephalogram (EEG). The AFz electrode was used as ground and two additional reference electrodes were placed on the left and right mastoids. To control for eye movements, two electrodes were placed on the outer canthi of the eyes, and one electrode was placed above the nasion. Impedances of electrodes were kept under 10 kΩ. The continuous EEG signal was amplified, digitized with a resolution of 1,000 Hz and pre-filtered with a notch filter at 50 Hz using a BrainAmp Standard amplifier (Brain Products GmbH, Gilching, Germany).

### Data processing

EEG signals were preprocessed and analyzed with the software Brain Vision Analyzer (version 2.1; Brain Products GmbH, Gilching, Germany). The signals were referenced to linked mastoids, high-pass filtered (0.2 Hz, 24 dB/octave), and corrected for eye movements based on Gratton et al. (1983).

#### Steady-state evoked potentials

**Figure 3:**
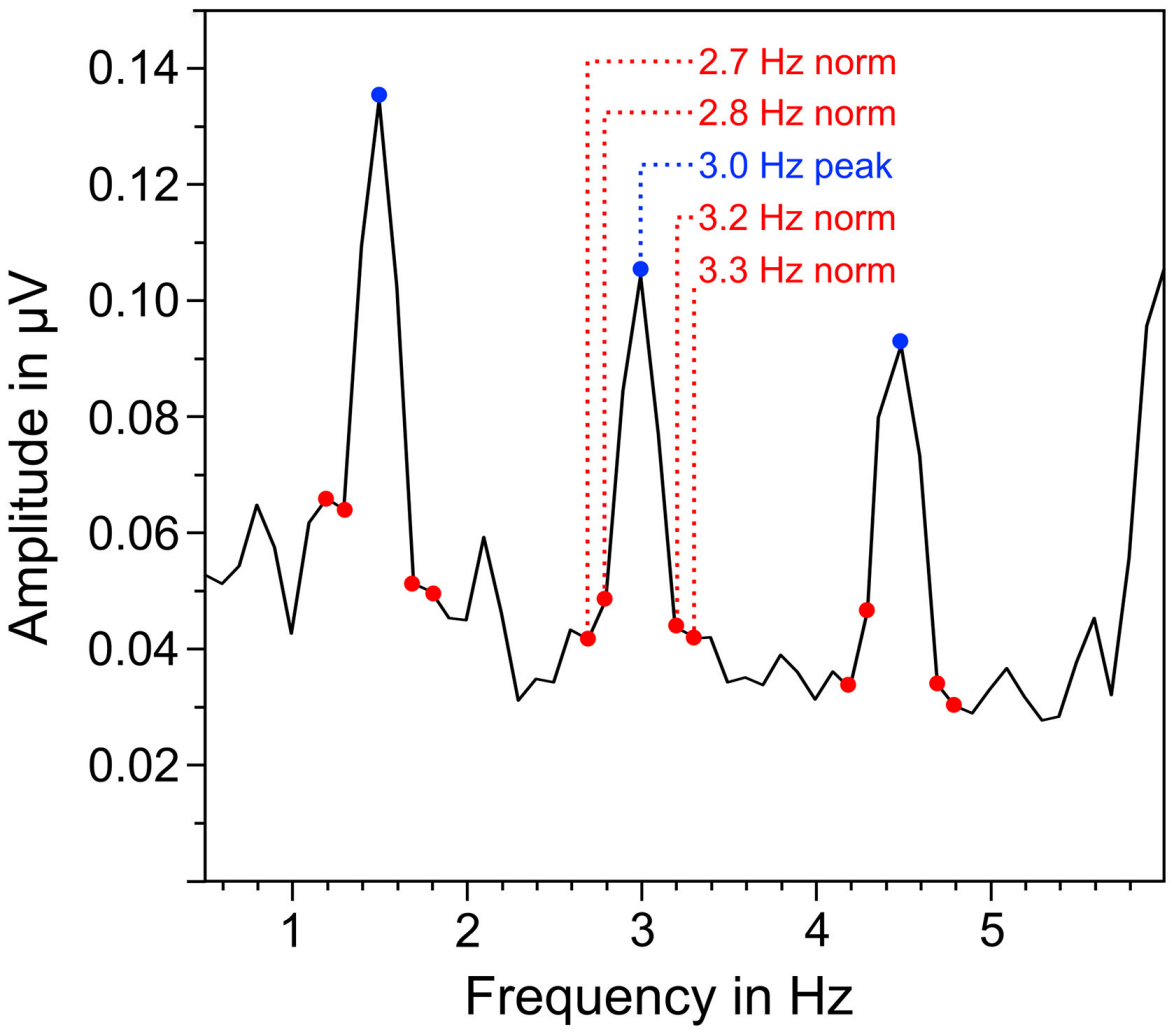
**Exemplary illustration of the normalization of neural oscillations at beat-related frequencies**. The mean amplitude of surrounding frequencies (marked in red) was subtracted from the amplitude at the corresponding beat-related frequency (marked in blue).

EEG signals of each trial were segmented in three different parts that corresponded to the different parts in the auditory stimuli: quadruple rhythm (4 s), 4:3 polyrhythm (4 s), and silent break (2.33 s; Figure 1B). The EEG signals during 2-min listening to the triple metronome before and after the experiment were segmented in 4 s segments. The first of those segments was removed to exclude ERPs related to the onset of the stimuli (Nozaradan et al., 2011). Segments with voltage steps above 120 μV/ms, or over 200 μV in a 200 ms time window were rejected (3.49%). Overall numbers of averaged segments for the different parts of the auditory stimuli were 111.8 for quadruple drum rhythms, 116.3 for 4:3 polyrhythms, 117.4 for silent periods (maximum *N* = 120, respectively), and 57.9 for the triple metronome (maximum *N* = 58). First, EEG segments were averaged across trials for each participant in the time domain. Signals of the five fronto-central electrodes (Fz, FC1, FCz, FC2, Cz) were then averaged, because SSEPs are predominant in this area (Nozaradan et al., 2011, 2012a,b; Tierney and Kraus, 2015; Stupacher et al., 2016). The averaged signals were finally transformed from the time domain into the frequency domain via discrete fast Fourier transformation (FFT) with a frequency resolution of 0.1 Hz and 10% overlapping Hanning windows. Amplitudes of beat-related frequencies (f_n_) in the frequency spectra were normalized with the mean amplitude of four surrounding frequencies (f_n_ − 0.3 Hz, f_n_ − 0.2 Hz, f_n_ + 0.2 Hz, f_n_ + 0.3 Hz; Figure 3). To assess whether SSEPs were elicited, the resulting normalized amplitudes were compared against zero in one-sample *t*-tests.

The SSEP analysis was focused on the first (f_1_) and second (f_2_) harmonic frequency corresponding to quarter and eighth notes (Stupacher et al., 2016). Mean of 2 and 4 Hz normalized spectral peaks were computed to evaluate SSEPs elicited by quadruple rhythms and 4:3 polyrhythms, and ongoing neural oscillations during silent periods. Accordingly, 1.5 and 3 Hz normalized spectral peaks were averaged to evaluate SSEPs elicited by 4:3 polyrhythms and the triple metronome, and ongoing neural oscillations during silent periods.

Beat-related neural oscillations during silent periods were additionally analyzed separately for correct and incorrect responses to the target stimuli. The average number of segments used to compute the frequency spectra was 52.0 for correct responses and 65.4 for incorrect responses. The remaining processing steps were identical to those mentioned above.

#### Time-frequency analysis

To explore how neural entrainment evolves with auditory stimuli, we additionally computed time-frequency plots based on the full trials. The 10.33 s segments included the quadruple rhythms (0–4 s), the 4:3 polyrhythms (4–8 s), and the subsequent silent periods without the target stimuli (8–10.33 s). Trials that included artifacts (voltage steps over 120 μV/ms, or over 200 μV in a 200 ms time window) were rejected, resulting in an average of 109.8 of 120 segments per participant. Segments were averaged in the time domain for each participant. The resulting signals of the five fronto-central electrodes were combined. Individual time-frequency plots for each participant were computed with continuous Morlet wavelet transformations (Morlet parameter c = 13, linear frequency steps from 1 to 8.5 Hz with a resolution of 0.125 Hz). The resulting plots were averaged across participants.

#### EMG control

To ensure that SSEPs were solely based on neural activity and not on beat-related body movements, EMG signals from the neck (splenius capitis), both arms (extensor digitorum communis), and both legs (musculus tibialis anterior) were transformed into the frequency domain and normalized in the same way as the EEG signals. Normalized spectral peaks of all five muscles were averaged. Spectral peaks related to the quadruple beat (2 and 4 Hz) and to the triple beat (1.5 and 3 Hz) were averaged. *T*-tests against zero on the resulting averaged spectral peaks indicated that there were no beat-related body movements (Table 1).

**Table 1:** **Results of ***t***-tests against zero on normalized mean EMG activity recorded from neck, arms, and legs for musicians and non-musicians combined**.

		**Quadruple rhythms**	**4:3 polyrhythms**	**Silent breaks**	**Triple metronome**
2 and 4 Hz normalized peaks	*t*_(25)_	1.29	1.18	−0.87	−0.99
	*p*	0.210	0.251	0.395	0.334
1.5 and 3 Hz normalized peaks	*t*_(25)_	−1.58	1.10	0.56	0.30
	*p*	0.126	0.281	0.583	0.769

*The non-significant results indicate that participants did not move their body in time with the stimuli*.

#### Event-related potentials

In addition to ongoing neural oscillations, we analyzed transient neural activity evoked by the target stimuli (i.e., ERPs). Trials that included artifacts in EEG or EOG channels (voltage steps over 120 μV/ms, or over 120 μV in a 200 ms time window) were rejected. After baseline corrections (−200 to 0 ms before stimulus onset) and low-pass filtering (20 Hz, 48 db/octave), ERPs corresponding to correct behavioral responses (*M* = 52.1 segments) and incorrect responses (*M* = 64.7 segments of a maximum of 120 segments, respectively) were averaged across trials in the three different *target time* conditions for each participant. The average number of segments per participant and *target time* was 17.4 (*SD* = 8.9) for correct responses and 21.6 (*SD* = 8.7) for incorrect responses. One participant was excluded from the analysis due to an insufficient number of artifact-free segments (<10 per *target time*). In three cases the number of segments per *target time* was below 4. Those values were replaced by the mean values of all participants in that condition. Similar to the analysis of SSEPs, amplitudes of fronto-central electrodes (Fz, FC1, FCz, FC2, Cz) were averaged. The ERP analysis was focused on P1, N1, and P2 components that are relevant for rhythm perception. The earlier components, such as P1 and N1, are typically attenuated with increasing predictability (Moberget et al., 2008; Lange, 2009; Bendixen et al., 2012; Sanabria and Correa, 2013; Stupacher et al., 2016), whereas the P2 component may be enhanced with increasing predictability (Sanabria and Correa, 2013). The local positive maximum between 30 and 90 ms after stimulus onset (P1), the local negative maximum between 50 and 180 ms after stimulus onset (N1), and the local positive maximum between 150 and 300 ms after stimulus onset (P2) was extracted for each individual participant. The mean amplitude of a time window of ±5 ms around these peaks was exported for statistical analyses.

## Results

### Behavioral results

**Figure 4:**
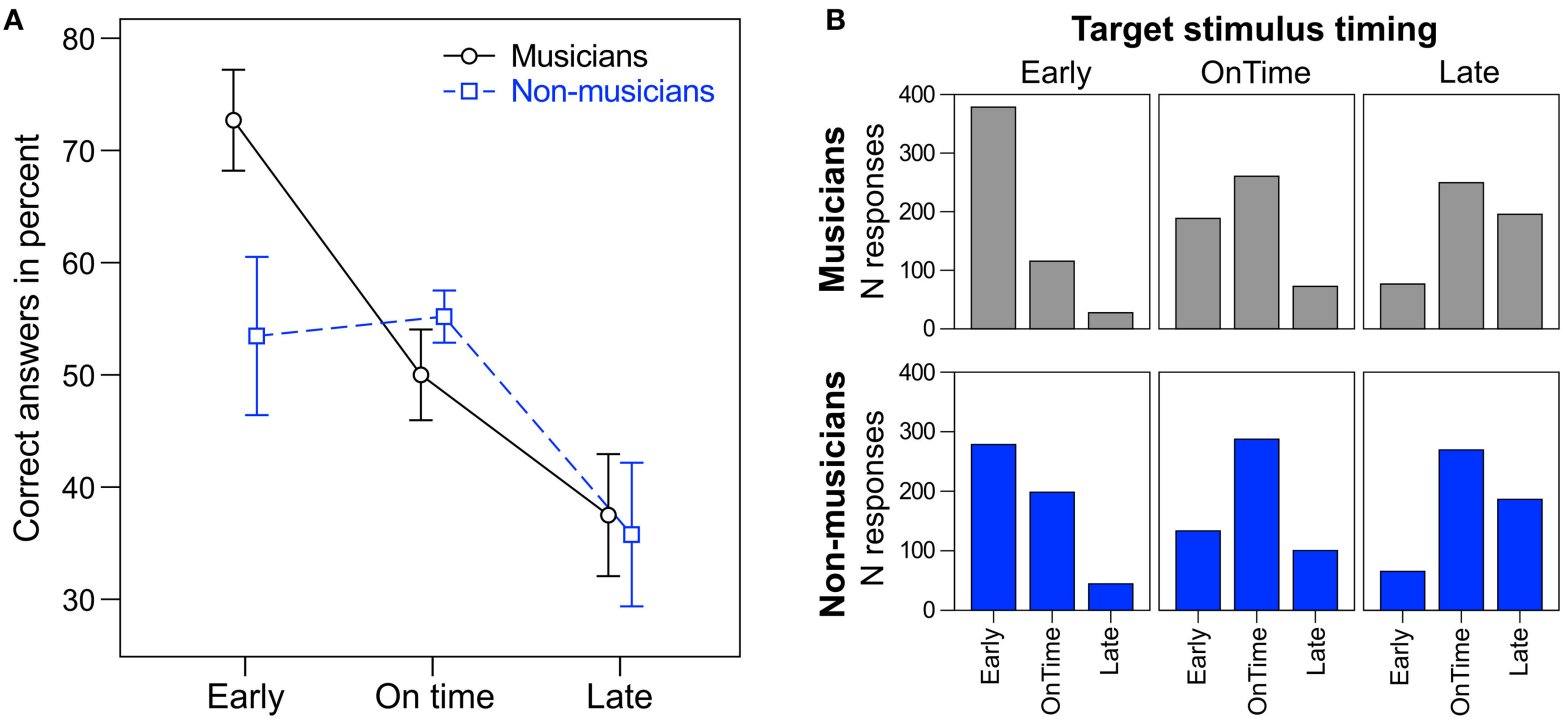
**(A)** Percentage of correct answers for musicians (round markers, solid black line) and non-musicians (square markers, dashed blue line). Error bars: ±1 SE. **(B)** Total number of responses (early, on time, late) to the different target stimuli (early, on time, late) for musicians (top panel, gray) and non-musicians (bottom panel, blue).

A repeated measures ANOVA on the percentage of correct answers with the within-subject factor *target time* (*early, on time*, and *late*) and the between-subject factor *musical expertise* revealed a main effect of *target time* [*F*_(2, 48)_ = 10.28, *p* < 0.001, η_*p*_^2^ = 0.30; Figure 4]. *Early* targets (*M* = 63%, *SD* = 23) were correctly identified more often than *on time* targets [*M* = 53%, *SD* = 12; *t*_(25)_ = 1.07, *p* = 0.049] and *late* targets [*M* = 37%, *SD* = 21; *t*_(25)_ = 3.51, *p* = 0.002]. Additionally, *on time* targets were identified correctly more often than *late* targets [*t*_(25)_ = 3.11, *p* = 0.005]. Musicians (*M* = 53%, *SD* = 6) tended to answer correctly more often than non-musicians [*M* = 48%, *SD* = 8; *F*_(1, 24)_ = 3.42, *p* = 0.077, η_*p*_^2^ = 0.16]. No interaction between *target time* and *musical expertise* was found [*F*_(2, 48)_ = 2.30, *p* = 0.112]. Individual *t*-tests showed that musicians answered correctly more often than non-musicians for *early* targets [*t*_(24)_ = 2.30, *p* = 0.030], but not for *on time* or *late* targets (*p*s > 0.27).

### Steady-state evoked potentials

**Figure 5:**
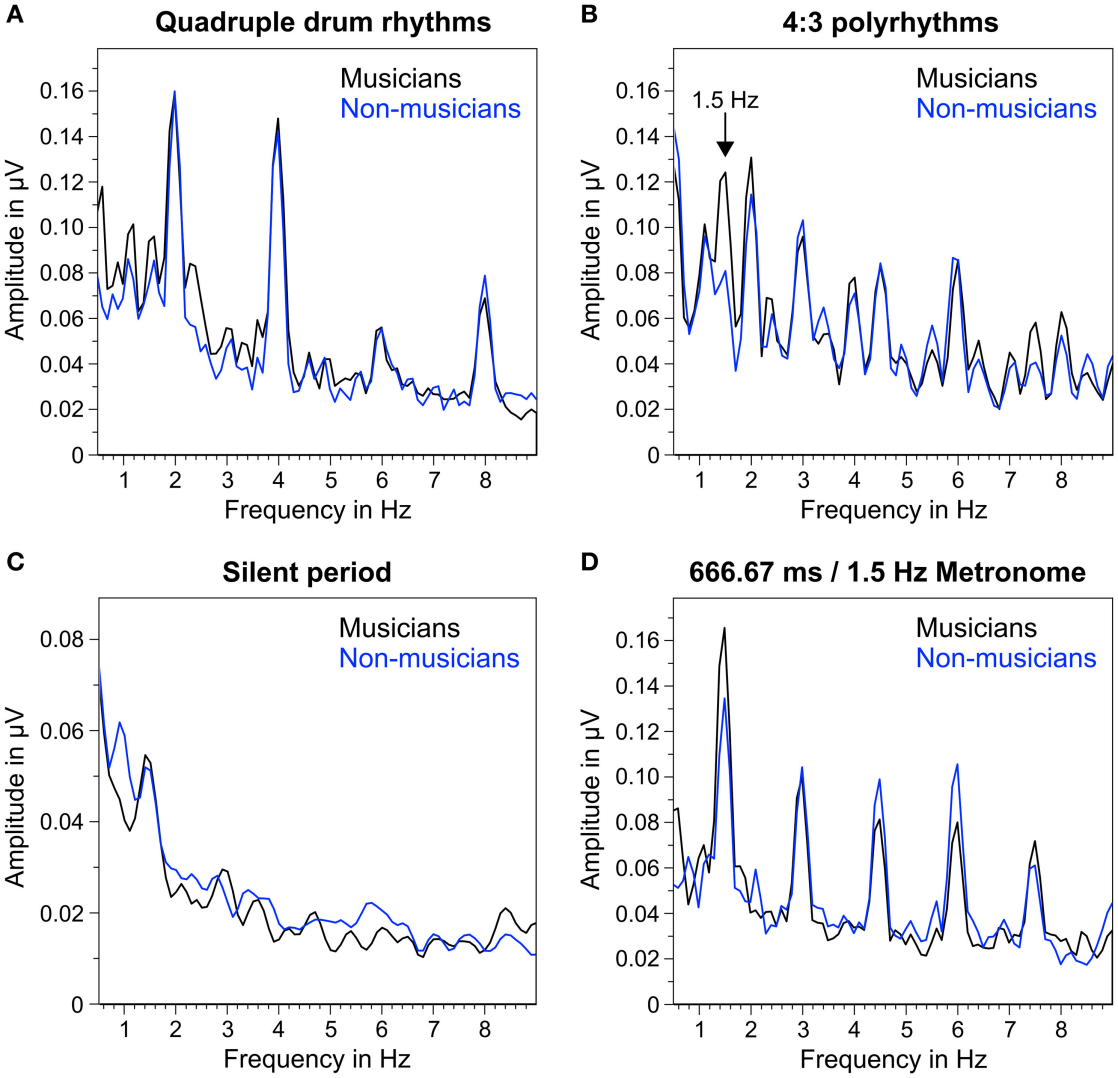
**Mean of musicians' (black lines) and non-musicians' (blue lines) frequency spectra of averaged fronto-central electrodes (Fz, FC1, FCz, FC2, Cz). (A)** During quadruple drum rhythms (0–4 s of each trial). **(B)** During 4:3 polyrhythms (4–8 s). **(C)** During the silent period before target stimulus presentation (8,000–10,333 ms). **(D)** During 2 min of listening to a metronome with a beat rate of 1.5 Hz at the beginning and end of the experiment.

Figure 5 shows the mean frequency spectra of participants' oscillatory brain activations during the different segments of the auditory stimuli. As expected, quadruple drum rhythms (Figure 5A) elicited clear amplitude peaks at 2 Hz (f_1_), 4 Hz (f_2_), and 8 Hz (f_4_), and a smaller peak at 6 Hz (f_3_). The same amplitude peaks (2, 4, 6, and 8 Hz) can be found in the frequency spectra during listening to the 4:3 polyrhythms (Figure 5B). Additionally, the 4:3 polyrhythms elicited amplitude peaks at 3 Hz (f_2_ of triple beat) and 4.5 Hz (f_3_ of triple beat) in both musicians and non-musicians. A clear peak at 1.5 Hz (f_1_ of triple beat) can only be seen in the frequency spectrum of musicians. The frequency spectra during silent periods show a similar pattern: At 1.5 Hz the amplitude peak of musicians is clearer than the one of non-musicians (Figure 5C). At 3 Hz a smaller and flatter peak can be found in musicians, but not in non-musicians.

The triple metronome (Figure 5D) with the same rate as the triple beat in the 4:3 polyrhythm (666.67 ms/1.5 Hz) elicited clear peaks at 1.5 Hz (f_1_), 3 Hz (f_2_), 4.5 Hz (f_3_), and 6 Hz (f_4_), suggesting that the frequency spectra of 4:3 polyrhythms represent a combination of SSEPs found with quadruple rhythms and triple metronome.

**Figure 6:**
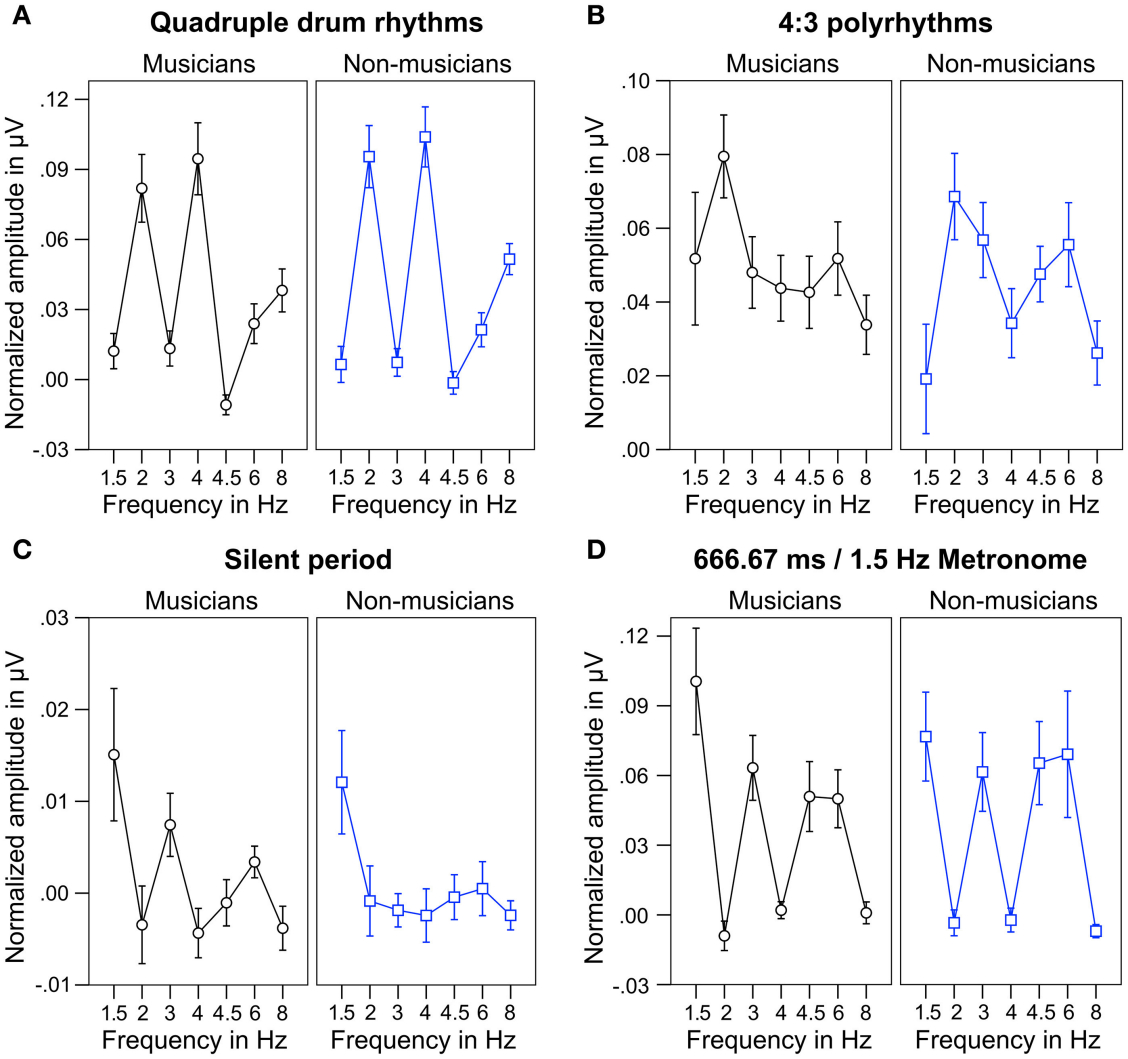
**Mean of musicians' (black lines, left panels) and non-musicians' (blue lines, right panels) normalized frequency spectrum amplitudes at quadruple-beat-related (2, 4, 6, 8 Hz) and triple-beat-related frequencies (1.5, 3, 4.5, 6 Hz). (A)** During quadruple drum rhythms (0–4 s of each trial). **(B)** During 4:3 polyrhythms (4–8 s). **(C)** During the silent period before target stimulus presentation (8,000–10,333 ms). **(D)** During 2 min of listening to a metronome with a beat rate of 1.5 Hz at the beginning and end of the experiment. Error bars: ±1 SE.

For statistical analyses, we computed the mean of normalized spectral peaks at 2 and 4 Hz to evaluate SSEPs elicited by the quadruple beat and spectral peaks at 1.5 and 3 Hz to evaluate SSEPs elicited by the triple beat. Figure 6 shows an overview of the SSEP results for the individual frequencies. *T*-tests against zero revealed that in both musicians and non-musicians quadruple-beat-related SSEPs were elicited during listening to quadruple rhythms and 4:3 polyrhythms (all *p*s < 0.001; Table 2). In both groups, triple-beat-related SSEPs were elicited during listening to 4:3 polyrhythms and the triple-beat metronome (all *p*s ≤ 0.001). In silent periods endogenous neural oscillations at triple-beat-related frequencies were found in musicians (*t* = 2.30, *p* = 0.040) but not in non-musicians (*t* = 1.75, *p* = 0.105). Independent samples *t*-tests on the amplitude of quadruple- and triple-beat-related SSEPs during listening to quadruple rhythms, 4:3 polyrhythms, and the triple-beat metronome revealed no difference between musicians and non-musicians (all *p*s > 0.3). The comparison of endogenous neural oscillations during silent periods showed a difference between musicians' and non-musicians' at the second harmonic [3 Hz; *t*_(24)_ = 2.39, *p* = 0.025], with higher normalized amplitudes for musicians (*M* = 0.007 μV, *SD* = 0.012) than for non-musicians (*M* = −0.002 μV, *SD* = 0.007). The strength of neural oscillations at the first harmonic frequency (1.5 Hz) did not differ between the two groups [*t*_(24)_ = 0.33, *p* = 0.746].

**Table 2:** **Results of ***t***-tests against zero on normalized spectral peaks at quadruple-beat-related (2 and 4 Hz) and triple-beat-related (1.5 and 3 Hz) frequencies**.

			**Quadruple rhythms**	**4:3 polyrhythms**	**Silent breaks**	**Triple metronome**
Musicians	2 and 4 Hz normalized peaks	*t*_(12)_	7.28	9.94	−1.80	−0.76
		*P*	<0.001^***^	<0.001^***^	0.098	0.462
	1.5 and 3 Hz normalized peaks	*t*_(12)_	2.30	5.12	2.30	4.90
		*p*	0.040^*^	<0.001^***^	0.040^*^	<0.001^***^
Non-musicians	2 and 4 Hz normalized peaks	*t*_(12)_	9.54	5.54	−1.37	−0.74
		*P*	<0.001^***^	<0.001^***^	0.195	0.473
	1.5 and 3 Hz normalized peaks	*t*_(12)_	1.33	4.12	1.75	4.44
		*p*	0.209	0.001^***^	0.105	0.001^***^

Significant values (^*^p < 0.05, ^***^p < 0.001) indicate the existence of SSEPs at the corresponding frequencies.

**Figure 7:**
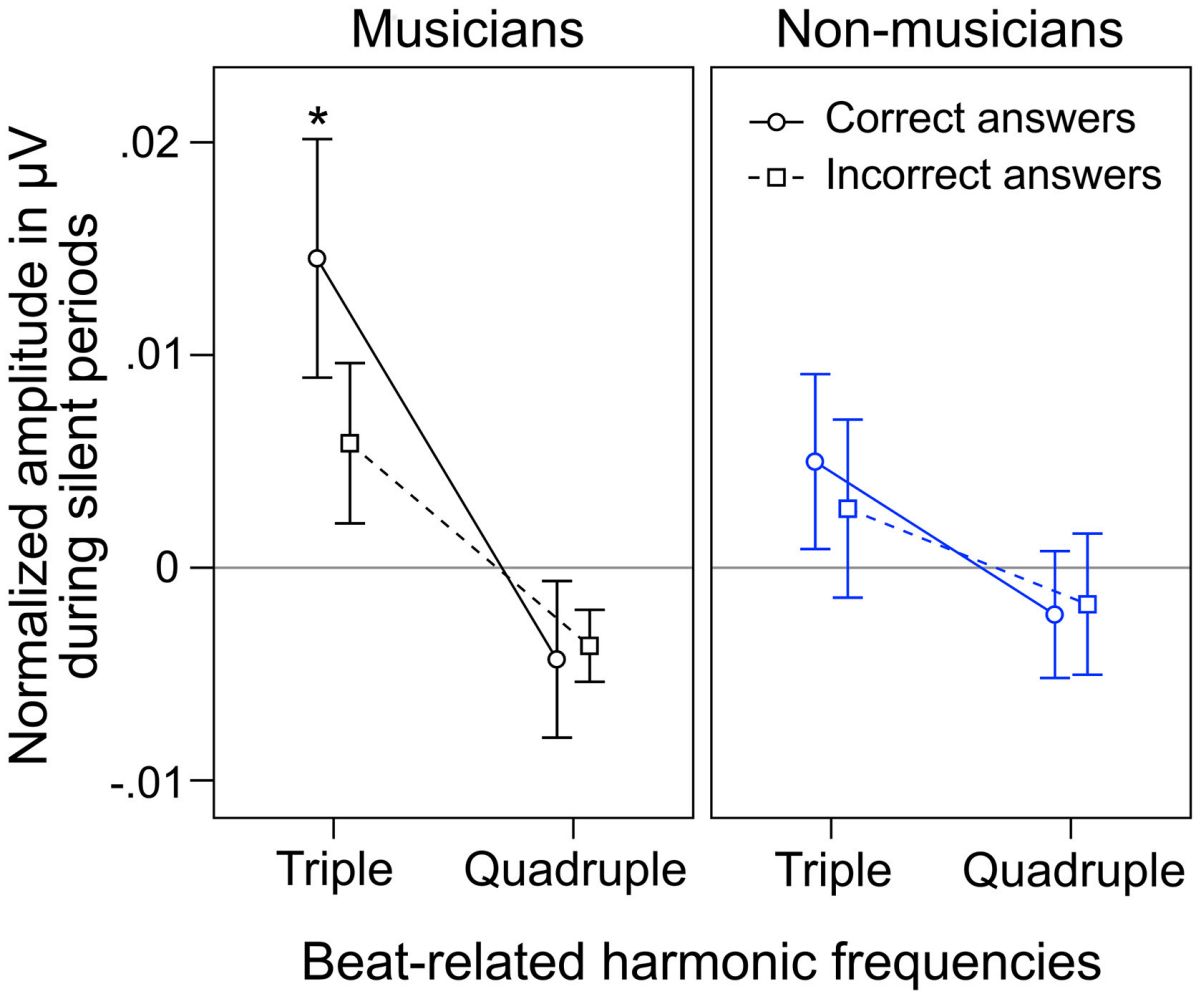
**Mean of musicians' (black lines, left panels) and non-musicians' (blue lines, right panels) normalized frequency spectrum amplitudes at quadruple-beat-related (2 and 4 Hz combined) and triple-beat-related frequencies (1.5 and 3 Hz combined) during silent periods before target stimulus presentation**. Focusing on the triple beat was more task-relevant than focusing on the quadruple beat. ^*^p < 0.05 in *t*-test against zero. Error bars: ±1 SE.

During silent periods, we additionally analyzed beat-related neural oscillations corresponding to correctly and incorrectly judged target stimuli. An ANOVA on normalized peak amplitudes during silent periods with the within-subject factors *beat-related frequencies* (*triple, quadruple*) and *answer* (*correct, incorrect*) and the between-subject factor *musical expertise* revealed a main effect of *beat-related frequencies* [*F*_(1, 24)_ = 10.81, *p* = 0.003, η_*p*_^2^ = 0.31] with higher amplitudes at triple-beat-related frequencies (1.5 and 3 Hz; *M* = 0.007 μV, *SD* = 0.016) compared to quadruple-beat-related frequencies (2 and 4 Hz; *M* = −0.003 μV, *SD* = 0.011; Figure 7). No other main effect or interaction was found (all *p*s > 0.18). Two separate ANOVAs, one for each *musical expertise* group, showed that the main effect of *beat-related frequencies* was mainly driven by musicians [*F*_(1, 12)_ = 7.41, *p* = 0.019, η_*p*_^2^ = 0.22, for musicians; *F*_(1, 12)_ = 3.44, *p* = 0.089, η_*p*_^2^ = 0.31, for non-musicians; Figure 7]. In both ANOVAs no main effects of *answer* and no interactions were found (all *p*s > 0.25).

In silent periods, we found a negative correlation between the amplitude of neural oscillations at triple and quadruple beat frequencies [*r*_(26)_ = −0.48, *p* = 0.014], indicating that high amplitudes at triple-beat-related frequencies were associated with low amplitudes at quadruple-beat-related frequencies. During listening to the 4:3 polyrhythms, the correlation between the amplitude of neural oscillations at triple and quadruple beat frequencies was not significant [*r*_(26)_ = −0.07, *p* = 0.752].

**Figure 8:**
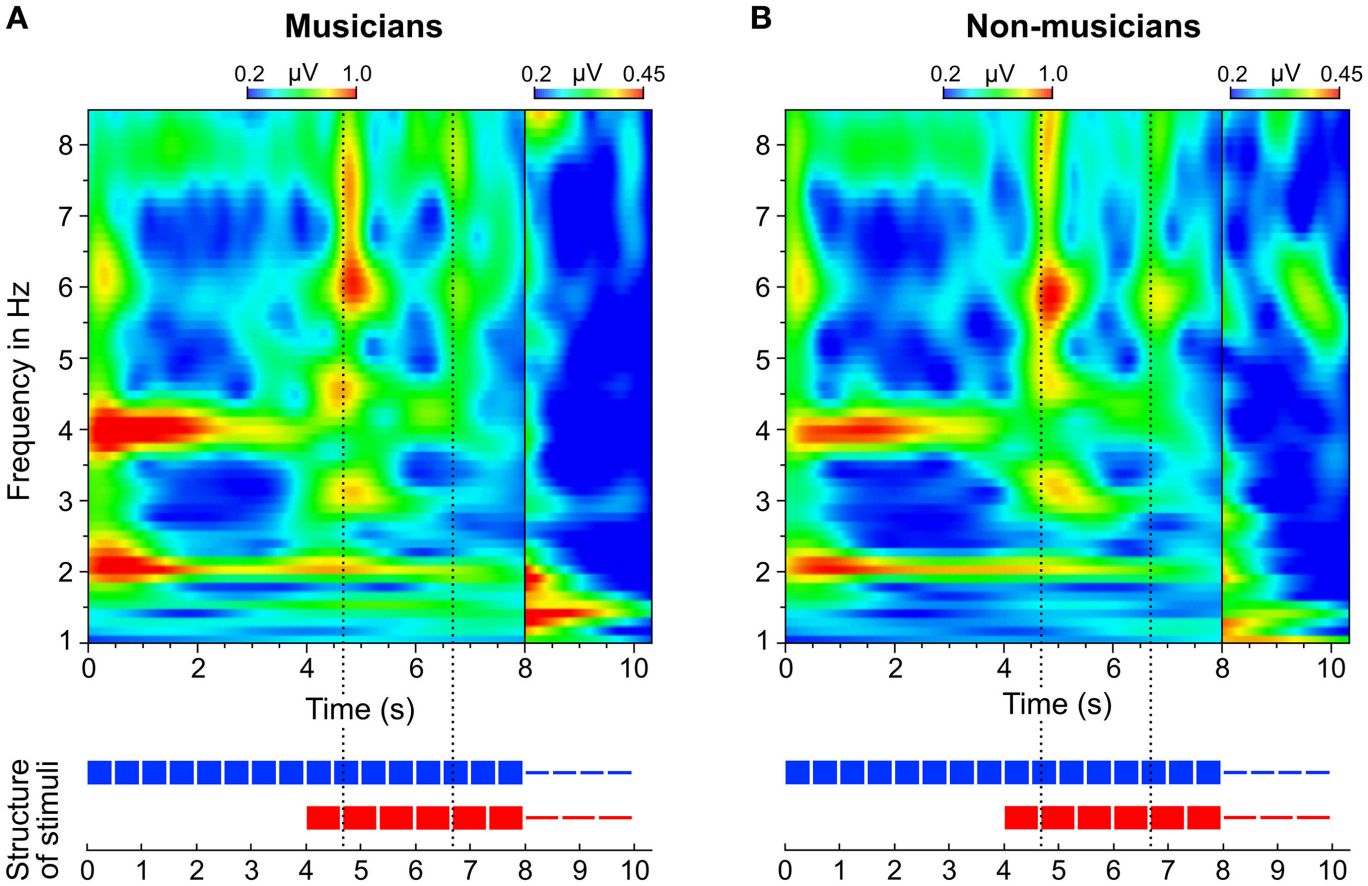
**Time-frequency plots of fronto-central electrodes (Fz, FC1, FCz, FC2, Cz) averaged over (A)** musicians and **(B)** non-musicians. Heatmaps represent the mean amplitude over all trials (0.2–1.0 μV during stimulus presentation and 0.2–0.45 μV during silent periods).

**Figure 9:**
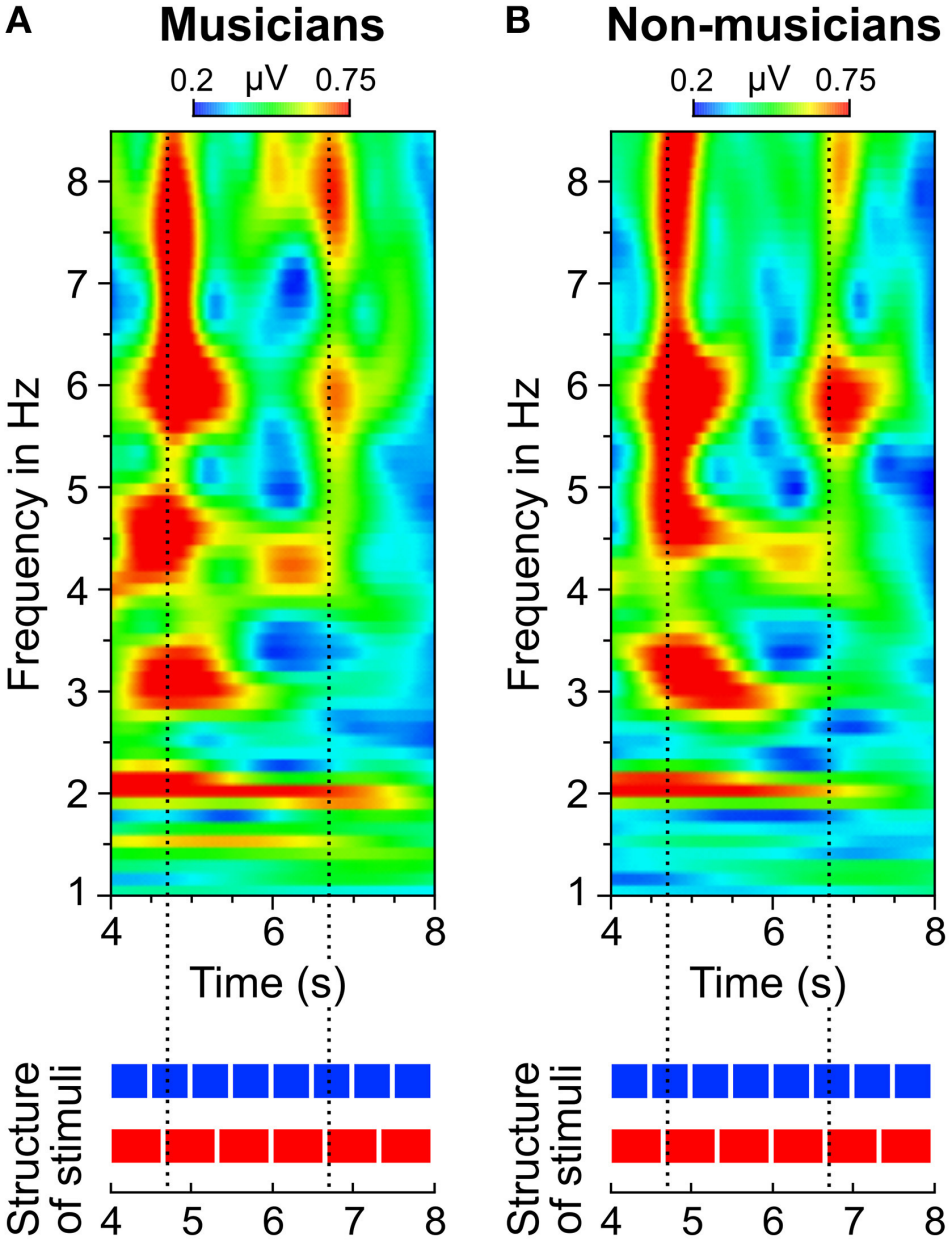
**Time-frequency plots of fronto-central electrodes (Fz, FC1, FCz, FC2, Cz) averaged over (A)** musicians and **(B)** non-musicians during listening to the 4:3 polyrhythm. Heatmaps represent the mean amplitude over all trials (0.2–0.75 μV).

In addition to the analysis of neural oscillations based on discrete FFTs, we computed time-frequency plots based on continuous wavelet transformations. Figure 8 shows two separate time-frequency plots for musicians and non-musicians and the corresponding segments of the auditory stimuli. A comparison between the plots reveals two main differences. (1) In contrast to non-musicians, musicians show ongoing neural oscillations at 1.5 Hz, starting with the 4:3 polyrhythm (see also Figure 9). (2) Compared to non-musicians, musicians have higher amplitudes at 1.5 and 3 Hz during the silent periods starting at second 8. In both plots, SSEPs at 2 and 4 Hz (f_1_ and f_2_ of quadruple beat) are clearly visible during the quadruple drum rhythms (0–4 s) and become weaker during 4:3 polyrhythms (4–8 s).

### Behavioral relevance of endogenous neural entrainment

A positive correlation between triple-beat-related neural oscillations (1.5 and 3 Hz) during silent periods and the number of correctly classified target stimuli (*early, on time, late*) indicates that participants with higher endogenous neural entrainment at task-relevant frequencies were better at recognizing the timing of the target stimulus [*r*_(26)_ = 0.47, *p* = 0.015]. However, bootstrapping of the correlation (*k* = 10,000 estimations with replacement; sample size *n* = 26) revealed that this correlation is not stable, as its 95% CI did include zero. To further evaluate the relationship between behavioral responses and endogenous neural oscillations, we computed two separate correlations for non-musicians [*r*_(13)_ = 0.68, *p* = 0.010] and musicians [*r*_(13)_ = 0.30, *p* = 0.327], indicating that the whole-sample correlation was mainly driven by non-musicians (for the significant correlation in non-musicians, bootstrapping (*k* = 10,000) revealed a 95% CI that did not include zero [0.38, 0.89]. The correlation between quadruple-beat-related SSEPs (2 and 4 Hz) and the number of correct answers was negative, but not significant [*r*_(26)_ = −0.29, *p* = 0.147].

### Event-related potentials

**Figure 10:**
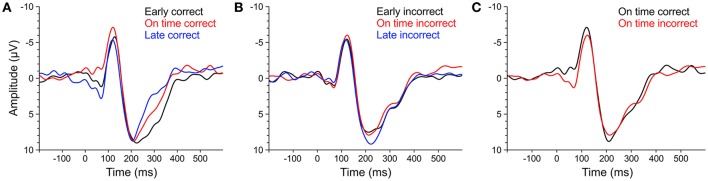
**ERPs of averaged fronto-central electrodes (Fz, FC1, FCz, FC2, Cz) elicited by ***early*** (black line), ***on time*** (red line), and ***late*** (blue line) target stimuli that were subsequently answered correctly (A)** or incorrectly **(B)**. **(C)** ERPs elicited by *on time* target stimuli that were subsequently answered correctly (black line) or incorrectly (red line). ERPs of musicians and non-musicians were combined, as there were no significant effects of *musical expertise* and no interactions between *musical expertise* and *target time*.

An overview of the ERP results is shown in Table 3. We will first present statistical analyses of ERPs corresponding to correct behavioral responses (ERP_cor_; Figure 10A) followed by analyses of ERPs corresponding to incorrect behavioral responses (ERP_incor_; Figure 10B).

**Table 3:** **Mean amplitudes in μV and locations in milliseconds of ERP components (P1, N1, P2) elicited by the target stimuli (early, on time, late) corresponding to correct and incorrect behavioral responses**.

**Answer**	**Component**	**Target time**	**Musicians**				**Non-musicians**			
			**Mean amplitude (μV)**	***SD* of amplitude**	**Mean location (ms)**	***SD* of location**	**Mean amplitude (μV)**	***SD* of amplitude**	**Mean location (ms)**	***SD* of location**
Correct	P1	Early	2.33	3.68	68	17	3.26	3.24	62	16
	P1	On time	0.71	1.90	59	20	−0.25	2.03	63	17
	P1	Late	3.02	3.33	64	14	4.01	3.31	64	17
	N1	Early	−6.86	4.67	122	17	−7.73	6.42	115	14
	N1	On time	−8.99	5.15	120	14	−7.54	3.31	117	12
	N1	Late	−6.49	5.44	116	11	−6.89	4.68	120	16
	P2	Early	12.31	5.69	225	28	10.07	5.29	229	30
	P2	On time	11.29	3.60	217	24	9.42	3.49	226	36
	P2	Late	10.84	4.65	209	21	9.48	4.07	199	20
Incorrect	P1	Early	1.83	2.70	64	13	2.21	1.98	63	17
	P1	On time	2.24	3.06	67	10	2.67	2.23	55	18
	P1	Late	1.90	2.04	64	16	1.22	1.60	62	17
	N1	Early	−6.33	4.10	115	19	−6.79	5.04	119	10
	N1	On time	−6.76	3.84	117	25	−7.66	4.50	119	18
	N1	Late	−5.81	5.12	110	21	−7.30	4.33	114	12
	P2	Early	11.89	4.38	218	28	8.06	3.40	224	36
	P2	On time	10.73	3.89	233	30	8.93	4.53	210	29
	P2	Late	12.47	4.17	217	29	8.82	4.82	232	25

We computed separate ANOVAs on the amplitudes and the locations of P1_cor_, N1_cor_, and P2_cor_ components with the within-subject factor *target time* (*early, on time, late*) and the between-subject factor *musical expertise*. For the amplitudes, a significant main effect of *target time* was only found for the P1_cor_ component [*F*_(2, 46)_ = 7.81, *p* = 0.001, η_*p*_^2^ = 0.25; Figure 10A]. Amplitudes of P1_cor_ evoked by *on time* targets (*M* = 0.21 μV, *SD* = 1.99) were lower than amplitudes of P1_cor_ evoked by *early* targets [*M* = 2.81, *SD* = 3.42; *t*_(24)_ = −3.35, *p* = 0.003] and *late* targets [*M* = 3.54, *SD* = 3.29; *t*_(24)_ = −4.69, *p* < 0.001]. No difference was found between amplitudes of *late* and *early* targets [*t*_(24)_ = 0.67, *p* > 0.5]. The effect of *musical expertise* and the interaction between *musical expertise* and *target time* on the P1_cor_ amplitude were not significant (*p*s > 0.4). For the amplitudes of the N1_cor_ and P2_cor_ components, the effects of *target time, musical expertise*, and the interactions between the two factors were not significant (all *p*s > 0.2). For the locations, a significant main effect of *target time* was only found for the P2_cor_ component [*early*: *M* = 227 ms, *SD* = 29; *on time*: *M* = 221 ms, *SD* = 31; *late*: *M* = 203 ms, *SD* = 21; *F*_(2, 46)_ = 4.86, *p* = 0.012, η_*p*_^2^ = 0.17]. The effect of *musical expertise* and the interaction between *musical expertise* and *target time* on the P2_cor_ location were not significant (*p*s > 0.4). For the locations of the N1_cor_ and P2_cor_ components, the effects of *target time, musical expertise*, and the interactions between the two factors were not significant (all *p*s > 0.08). Separate ANOVAs on the amplitudes of P1_incor_, N1_incor_, and P2_incor_ components showed no significant main effects of *target time* or *musical expertise*, and no interactions (all *p*s > 0.05).

In an additional analysis we focused on *on time* targets only and compared the amplitude of ERPs related to correct and incorrect behavioral responses. An ANOVAs on P1 amplitudes revealed a main effect of correct (*M* = 0.21, *SD* = 1.99) vs. incorrect (*M* = 2.46, *SD* = 2.61) *on time* targets [*F*_(1, 23)_ = 17.44, *p* < 0.001, η_*p*_^2^ = 0.43; Figure 10C], no significant effect of *musical expertise* and no significant interaction (*p*s > 0.2). ANOVAs on N1 and P2 amplitudes showed no significant effects of correct vs. incorrect responses, no effects of *musical expertise* and no interactions (*p*s > 0.15).

A visual inspection of the time-frequency plots (Figures 8, 9) suggests that in both musicians and non-musicians, the second onset of the triple woodblock (equivalent to the second beat of the triple meter) elicited a prominent event-related potential. A less pronounced event-related potential can be found at the time of the fifth onset of the triple woodblock. An ANOVA on the N1 amplitude showed that the N1 elicited by the second triple beat (*M* = −4.28, *SD* = 2.01) was more pronounced than the N1 elicited by the fifth triple beat [*M* = −1.93, *SD* = 2.09; *F*_(1, 23)_ = 39.24, *p* < 0.001, η_*p*_^2^ = 0.63]. N1 amplitudes did not differ between musicians and non-musicians and did not interact with musical expertise (both *p*s > 0.6). Two additional ANOVAs on P1 and P2 amplitudes showed no significant differences between second and fifth beat, musicians and non-musicians, and no interaction (all *p*s > 0.05). This *post-hoc* analysis is a first step toward a more systematic characterization of neural entrainment over time.

## Discussion

Empirical findings, computational models, hypotheses and theories suggest that the entrainment of neural oscillations to musical rhythm enables us to disentangle and organize temporal structures and to perceive a steady beat (Large et al., 2015). The current study adds to this growing body of research. By using auditory stimuli that changed from quadruple isorhythms to 3-over-4 polyrhythms, we showed that neural oscillations evolve with changing and overlaying rhythmic frameworks. By adapting a previous design in which the stimuli were interrupted by silent breaks (Stupacher et al., 2016), we provided evidence that *endogenous* neural oscillations persist without external stimulation. By combining EEG recordings and behavioral data, we assessed the relevance of *endogenous* neural entrainment for rhythm processing. And finally, by comparing data of musicians and non-musicians we gained first insights into whether beat-related neural oscillations are modulated by musical expertise and in which ways.

The neural resonance theory of pulse and meter proposes that neural oscillations entrain to the temporal patterns of music (Large and Kolen, 1994; Large and Jones, 1999; Large, 2008). Neural entrainment requires a neural oscillator, a periodic external stimulus, and the synchronization between the two (Thut et al., 2011; Large et al., 2015). All of these assumptions were met in the current study. The time-frequency plots in Figures 8, 9 show the evolvement of neural entrainment over time during listening to the different parts of the auditory stimuli. As proposed by the neural resonance theory of pulse and meter (Large and Kolen, 1994; Large and Jones, 1999), the plots show that neural oscillations entrained to the different metric hierarchies of the rhythms. During the isorhythmic quadruple beat (0–4 s), neural oscillations of both, musicians and non-musicians are strongest at 2 and 4 Hz (quarter-note and eighth-note level of quadruple beat, respectively). During the subsequent 4:3 polyrhythm (4–8 s) the patterns change: Neural oscillations at 2 and 4 Hz become weaker and additional oscillations at 1.5 and 3 Hz emerge. This observation of fine-resolved oscillatory dynamics is supported by more commonly used analyses of the EEG signals' frequency spectra (Table 2, Figures 5, 6), which showed that quadruple rhythms elicited SSEPs at 2 and 4 Hz and that 4:3 polyrhythms additionally elicited neural oscillations at 1.5 and 3 Hz.

Figure 6 shows that the quadruple rhythms (Figure 6A) and the triple-beat metronome (Figure 6D)—the two different parts of the 4:3 polyrhythm—resulted in contrasting SSEP patterns. Each pattern reflects the metric hierarchies of the corresponding auditory stimulus. SSEPs of the 4:3 polyrhythm (Figure 6B) appear to be a combination of quadruple- and triple-beat-related SSEPs. This is an interesting finding, because participants were instructed to focus on the triple beat of the polyrhythm. Additionally, the behavioral task made it advantageous for the participants to only track the triple beat. If the two different parts of the polyrhythm could be processed independently by parallel timekeepers, participants should have focused their attention on the triple-beat part. Consequently, with parallel timekeepers the frequency pattern of neural oscillations during the polyrhythm should reflect the triple-beat part and be similar to the frequency patterns during the silent period (Figure 6C) and the triple-beat metronome (Figure 6D). However, in both musicians and non-musicians, the most pronounced SSEP can be found at the quadruple-beat-related frequency of 2 Hz. Additionally, the frequency spectra of both musicians and non-musicians show a prominent peak at 6 Hz—the only frequency shared by the quadruple and triple beat (Figures 5B, 6B). Importantly, the frequency spectra of the sound envelopes of the polyrhythms (Figure 2B, bottom panel) do not show such a prominent peak at 6 Hz. In line with previous findings, these results suggest that polyrhythms, at least up to a certain complexity, are not processed by parallel timekeepers but rather within a shared rhythmic framework (e.g., Deutsch, 1983; Klapp et al., 1985, 1998; Jagacinski et al., 1988; Summers and Kennedy, 1992; Summers et al., 1993; Jones et al., 1995; Peper et al., 1995; Klein and Jones, 1996; Krampe et al., 2000; Kurtz and Lee, 2003). Further evidence for a shared framework comes from the time frequency plots that revealed a prominent ERP elicited by the second onset of the triple-beat woodblock and a less prominent ERP elicited by the fifth triple-beat onset (marked by dotted lines in Figures 8, 9). The second and fifth onsets of the triple beat woodblock are the first onsets that lie outside the metric grid of the preceding quadruple beat (in the first and second bar of the 4:3 polyrhythm, respectively). Thus, these onsets are less expected than the others, because they violate the previously established metric structure. At first glance this finding seems to contradict the assumption of a shared rhythmic framework. However, as both the amplitudes in the time-frequency plots and the amplitudes of the N1 ERP components indicate that the second onset of the triple beat woodblock elicited more prominent responses than the fifth onset, it can be concluded that the fifth onset was more expected than the second onset. This finding suggests that the triple beat is gradually integrated into the rhythmic framework of the quadruple beat. Consistent with this interpretation, the third and sixth onset of the triple beat woodblock did not elicit an observable ERP.

In general, the finding of evolving neural oscillations that entrain to rhythmic stimuli with changing and overlaying rhythmic frameworks shows that the analysis of SSEPs provides a useful tool for the investigation of polyrhythm processing. Future EEG or EMG experiments could use the frequency-tagging approach to investigate whether more complex polyrhythms are processed differently (e.g., in parallel streams).

Besides neural entrainment to physically present acoustic rhythms, we recorded and analyzed *endogenous* neural entrainment during the silent periods between the presentations of rhythmic stimuli and target stimuli (Figure 1B). In musicians, the pattern of *endogenous* neural oscillations (Figure 6C, left panel) resembles the pattern observed during listening to the triple-beat metronome (Figure 6D, left panel). In non-musicians (Figure 6C, right panel), oscillations at 1.5 Hz stand out. The normalized peak amplitude at 1.5 Hz for both groups combined was larger than zero [*t*_(25)_ = 3.02, *p* = 0.006], indicating that *endogenous* neural oscillations at this frequency persisted throughout silent periods. As expected, these findings suggest that participants successfully imagined that the triple beat continues during silent periods. In line with this conclusion, Figure 7 shows that during the silent periods, task-relevant *endogenous* neural oscillations at triple-beat-related frequencies (1.5 and 3 Hz) were more pronounced than task-irrelevant oscillations at quadruple-beat-related frequencies (2 and 4 Hz). This is an important finding, as it suggests that neural oscillations during silent periods are not representing a “washing out” effect of the previous stimulation. If the endogenous neural oscillations were based on such a “washing out” effect, amplitudes at triple-beat- and quadruple-beat-related frequencies should be equally high.

The finding of beat-related neural oscillations that persist without external stimulation indicate that neural entrainment to music is not only driven by bottom-up processes, but also by top-down processes and *endogenous* oscillatory networks. Thus, our results support conclusions of a previous study using the silent break design (Stupacher et al., 2016), and studies showing that beat-related neural oscillations are elicited even if the fundamental beat frequency is not present in the acoustic signal (Nozaradan et al., 2012a, 2016; Large et al., 2015). In contrast to our previous study (Stupacher et al., 2016), which did not show persisting beat-related neural oscillations after participants listened to a drum rhythm, the current study provides direct evidence for *endogenously* created neural oscillations in a listening task without overt beat-related movement. A plausible reason for this discrepancy is that the instructions lead to different mental imagery states. Part of the task of the current study was to try to imagine that the triple beat continues during the silent period, whereas in Stupacher et al. (2016), participants were not instructed or explicitly motivated to imagine that the beat goes on after the stimulus stopped. By explicitly instructing participants to imagine that the stimulus continues, the current experiment intensified the recruitment of top-down processes, probably leading to strengthened *endogenous* neural entrainment.

Additional evidence for *endogenous* neural entrainment comes from the analysis of ERPs elicited by target tones that were presented after the silent periods (Figures 1B,C). For correctly identified target stimuli, the P1 amplitude elicited by targets presented on the triple beat timing grid (*on time*) was lower than the P1 amplitude elicited by *too early* and *too late* targets. We interpret the lower P1 amplitude as reflecting higher prediction accuracy because correctly identified *on time* targets evoked significantly lower P1 components than incorrectly identified *on time* targets. Support for this interpretation comes from a study by Moberget et al. (2008) who showed an enhancement of P1 amplitudes elicited by aperiodic stimuli (tones with mixed 650, 800, and 950 ms inter-stimulus-intervals) compared to periodic stimuli (fixed 800 ms inter-stimulus-interval) in healthy control participants. The functional relevance of the ERP data for timing judgements is supported by the finding that P1 amplitudes and P2 latencies were only affected by *target time* when the subsequent answer in the behavioral task was correct. How musical imagery relates to the ERP findings, why the amplitudes of N1 and P2 components were not modulated by the *target time*, how the effect of *target time* on P2 latencies can be explained, and what the underlying mechanisms of the P1 modulation could be remains a matter of future work.

By including a behavioral task, we were able to assess the functional relevance of *endogenous* neural entrainment (i.e., beat-related neural oscillations in silent periods) for rhythm and beat perception. Participants correctly identified the timing of the target stimulus (too early, on time, or too late) in about 51% of the cases (33.3% chance level), indicating that the task was not too easy and not too hard. Musicians tended to give more correct answers than non-musicians. In line with this finding, Rammsayer and Altenmüller (2006) showed that musical training can increase the sensitivity to deviations from regular beats. Interestingly, the difference between musicians and non-musicians was mainly driven by responses to *early* target stimuli (Figure 4). Overall, correct identification was highest for *early* and lowest for *late* targets. *Late* targets were often perceived as *on time* targets (Figure 4B). This results was unexpected, as in comparable conditions of a study by Barnes and Jones (2000; e.g., Table 2, 600 ms standard duration) correct identifications of shorter, same, or longer intervals compared to a context sequence were more symmetrically distributed. When comparing the duration of two time intervals, the preceding context rhythm can affect the temporal judgements (e.g., Barnes and Jones, 2000; McAuley and Jones, 2003). How the context of polyrhythms affects temporal judgements of a subsequent target tone remains to be investigated in greater detail.

For the whole sample of participants, we could show that *endogenous* neural entrainment improves fine-grained temporal processing, as indicated by a positive correlation between the number of correctly identified target stimuli and the amplitude of neural oscillations at 1.5 and 3 Hz during silent periods. This finding suggests that listeners use dynamic temporal patterns in form of entrained neural oscillations to process musical rhythms and to predict rhythmic events. In a recent study, Nozaradan et al. (2016), came to similar conclusions by showing that SSEPs during listening to an unsyncopated rhythm positively correlate with synchronization accuracy while tapping in time with the beat of the same rhythm. In the same study the authors computed a measure of *endogenous* neural entrainment by subtracting the SSEP amplitude during listening to the unsyncopated rhythm from the SSEP amplitude during listening to a syncopated rhythm. Comparable to the directly measured *endogenous* neural entrainment in the current study, the more indirect measure computed by Nozaradan et al. (2016) was associated with better temporal predictions.

To date, most neuroimaging studies on neural entrainment to music neglected the fact that musical expertise shapes how we perceive the temporal structures of music. In the current study, the comparison between musically trained and untrained participants revealed two main findings.

First, neural entrainment did not differ between musicians and non-musicians during listening to musical rhythms with low (triple metronome), medium (isometric quadruple rhythm), and high (4:3 polyrhythm) rhythmic complexities. These null results are intriguing because various behavioral and neuroimaging studies have shown that musical training sharpens temporal processing (e.g., Drake and Botte, 1993; Rammsayer and Altenmüller, 2006; van Vugt and Tillmann, 2014), improves sensorimotor synchronization accuracy and stability (e.g., Franek et al., 1991; Drake et al., 2000a,b; Krause et al., 2010; Repp, 2010; Baer et al., 2013; Cameron and Grahn, 2014; Baer et al., 2015), deepens the comprehension of metrical structures (e.g., Drake et al., 2000a,b; Brochard et al., 2003; van Zuijen et al., 2005; Geiser et al., 2010), modulates patterns of neural activation during rhythm perception, and strengthens neural auditory-motor coupling (e.g., Haueisen and Knösche, 2001; Bangert and Altenmüller, 2003; Drost et al., 2005; Haslinger et al., 2005; Vuust et al., 2005; D'Ausilio et al., 2006; Bangert et al., 2006; Lahav et al., 2007; Grahn and Rowe, 2009; Stupacher et al., 2013). The question arises as to why neural entrainment during stimulus presentation did not differ between musicians and non-musicians, although all of the aforementioned advantages of musical training seem to be relevant for entrainment processes.

To answer this question we should first take a look at the second main finding from the comparison of musical trained and untrained participants: *Endogenous* neural entrainment, as measured in silent periods, was stronger in musicians compared to non-musicians. This effect was shown by multiple different analyses. First, the amplitude of *endogenous* neural oscillations at triple-beat-related frequencies was significantly larger than zero in musicians but not in non-musicians. Second, in musicians, the amplitude of *endogenous* neural oscillations at triple-beat-related frequencies was significantly larger than the amplitude of task-irrelevant oscillations related to the quadruple beat (Figure 7). In non-musicians, this effect only showed a slight tendency in the same direction. Third, in musicians, the pattern of *endogenous* neural oscillations during silent periods (left panel of Figure 6C) resembled the pattern of neural oscillations elicited during listening to the triple metronome (left panel of Figure 6D). In contrast, the pattern of non-musicians (right panel of Figure 6C) lacked *endogenous* neural oscillations at frequencies other than 1.5 Hz. Consistent with this observation, the difference of *endogenous* neural entrainment between musicians and non-musicians was mainly driven by the strength of neural oscillations at 3 Hz—the second harmonic of the task-relevant triple beat. Fourth, the time-frequency plots of Figure 8 showed that *endogenous* neural oscillations (8–10 s) at triple-beat-related frequencies (1.5 and 3 Hz) were more pronounced in musicians compared to non-musicians. And finally, in accordance with the results of the neural entrainment analyses, behavioral results showed that musicians tended to be better at correctly identifying the timing of the target stimulus presented after the silent period.

To sum up the effects of musical expertise, analyses of ongoing neural activity and behavioral results suggest that *endogenous* neural entrainment during silent periods was stronger in musicians compared to non-musicians. In contrast, no difference of neural entrainment between the two expertise groups was found during stimulus presentation. How can we explain this divergence? The *endogenous* neural entrainment measured during silent periods is mainly driven by top-down processes, as participants had to imagine the triple beat without any external stimulation. In contrast, the neural entrainment measured during listening to the rhythms reflects a combination of top-down and bottom-up processes (Nozaradan et al., 2016; Stupacher et al., 2016), whereby the bottom-up component is driven by the acoustic input. During listening to music as well as during music production, hard-wired bottom-up sensation and experience-based top-down perception are interdependent. For example, one and the same rhythmic pattern can be interpreted in different ways depending on the metric framework the listener uses to structure the pattern (Honing, 2012). The different interpretations can be a result of different listening experiences, musical training backgrounds, or other individual factors. When briefly exposed to music of a foreign culture (2 weeks), infants, in contrast to adults, developed a culture-specific responsiveness and were able to differentiate culture-specific rhythmic patterns (Hannon and Trehub, 2005). Put another way, long time exposure to culture-specific music can narrow the types of musical patterns that we can effortlessly process. The stronger *endogenous* neural entrainment in musicians might also relate to better musical imagery abilities of musicians or musically engaged individuals compared to less musically trained or engaged individuals (e.g., Aleman et al., 2000; Jakubowski et al., 2016). Although top-down processes are crucial for beat and meter perception, our findings suggest that the amplitude of *endogenous* neural oscillations is lower than the amplitude of bottom-up driven neural oscillations. Thus, musical expertise might strengthen top-down driven *endogenous* neural entrainment during rhythm perception, but hard-wired bottom-up processes that do not differ between musicians and non-musicians might cover this effect.

In conclusion, the current study provides new evidence showing that neural oscillations at beat-related frequencies persist after the external acoustic input stopped. This finding indicates that besides bottom-up stimulus-driven processes, neural entrainment to music involves *endogenously* created neural oscillations related to higher order cognitive processes that enable us to predict what and when sounds are likely to occur. The comparison of musicians and non-musicians suggests that those *endogenously* created neural oscillations are strengthened by musical expertise. Moreover, our study demonstrates that neural entrainment is crucial for rhythm perception and processing, even when listening to stimuli with complex rhythmic frameworks such as polyrhythms.

## Author contributions

JS and MW designed the study, managed data collection, analyzed the data, and wrote the manuscript. GW designed the study and wrote the manuscript.

### Conflict of interest statement

The authors declare that the research was conducted in the absence of any commercial or financial relationships that could be construed as a potential conflict of interest.
